# Robot-assisted navigation combined with a double reverse traction reduction frame for retrograde intramedullary nailing of extra-articular distal femoral fractures: a retrospective comparative cohort study

**DOI:** 10.3389/fmed.2026.1929866

**Published:** 2026-10-19

**Authors:** Bo Wu, Bing Du, Yuhang Liu, Xianjie Ai, Chengcheng Zhang, Hongfei Qi, Zhong Li, Xiaye Lv, Ming Li

**Affiliations:** 1 Lower Extremity Division, Orthopedic Trauma Department, Honghui Hospital, Xi’an Jiaotong University, Xi’an, China; 2 Department of Hematology and Oncology, Honghui Hospital, Xi’an Jiaotong University, Xi’an, China

**Keywords:** distal femoral fracture, double reverse traction reduction frame (DRTR), retrograde intramedullary nailing, robot-assisted surgery, surgical workflow optimization

## Abstract

**Objective:**

To compare the perioperative, radiographic, and functional outcomes associated with an integrated surgical workflow combining robot-assisted navigation with a double reverse traction reduction frame vs. conventional manual traction.

**Methods:**

This retrospective comparative cohort study was conducted on 58 patients (AO/OTA 33-A1 or 33-A2) with distal femoral fractures treated between January 2021 and December 2024. Patients were divided into a robot-assisted double reverse traction reduction group (RDR group, *n* = 26) and a conventional manual traction group (CMT group, *n* = 32). All patients underwent retrograde intramedullary nailing. The RDR group received robot-assisted guidewire positioning combined with continuous traction-frame-assisted fracture reduction, whereas the CMT group underwent conventional manual traction reduction. Perioperative parameters, radiographic outcomes, functional scores, and complications were analyzed.

**Results:**

The groups were similar in the measured baseline demographic and fracture characteristics, including AO/OTA subtype. Compared with the CMT group, the RDR group showed significantly reduced operative time, decreased intraoperative blood loss, fewer fluoroscopic acquisitions, smaller residual coronal and sagittal angulation, and shorter fracture healing time. At the 12-month follow-up, knee flexion angle and Hospital for Special Surgery (HSS) knee score were significantly higher in the RDR group. No significant difference in complication rates was observed between groups.

**Conclusion:**

In this retrospective comparative cohort, the integrated workflow was associated with shorter operative time, lower intraoperative blood loss, fewer fluoroscopic acquisitions, improved postoperative alignment, and better 12-month knee function compared with conventional manual traction.

## Introduction

Distal femoral fractures are complex injuries that present significant challenges in orthopedic trauma surgery, particularly in elderly and osteoporotic patients. Due to the anatomical characteristics of the distal femur and the tendency toward comminution, achieving accurate reduction and stable fixation remains technically demanding. Retrograde intramedullary nailing is widely used as a minimally invasive fixation method; however, its success strongly depends on precise entry point localization, and maintenance of fracture alignment during nail insertion (1, 2).

Currently, the primary internal fixation methods include locking plate systems and intramedullary nail techniques (3, 4). Compared with plate fixation, retrograde intramedullary nailing provides superior biomechanical stability, higher fracture healing rates, and lower complication rates, and has therefore been widely adopted in clinical practice (5–9). However, this technique remains technically demanding, particularly in achieving accurate entry point positioning and maintaining stable reduction during nail insertion (10).

Despite its advantages, retrograde intramedullary nailing is still largely dependent on manual traction and fluoroscopy-guided reduction in the conventional surgical workflow. This traditional approach is associated with several limitations, including variability in reduction quality, dependence on surgeon experience, difficulty in maintaining continuous traction, and increased intraoperative radiation exposure due to repeated fluoroscopic imaging (11). These limitations highlight the need for a more standardized and reproducible surgical workflow for distal femoral fracture management.

In recent years, orthopedic robotic navigation systems have been introduced to improve the precision of implant placement by enabling accurate preoperative planning and intraoperative guidewire positioning. Studies have shown that robotic assistance significantly improves accuracy and reduces radiation exposure during intramedullary nail procedures and screw fixation in lower extremity fractures (12, 13). In parallel, the double reverse traction reduction frame has been introduced to provide continuous mechanical traction during fracture reduction, with previous studies reporting potential advantages over manual traction or traction-table techniques in selected lower-extremity fractures (14). However, robotic navigation and mechanically assisted traction have generally been evaluated separately, and their coordinated application within a single workflow for retrograde intramedullary nailing of distal femoral fractures remains insufficiently studied.

We hypothesized that integrating robot-assisted navigation with continuous mechanical traction could address two complementary procedural challenges in distal femoral fracture fixation: guidewire planning and positioning, and maintenance of fracture alignment during retrograde intramedullary nailing. Coordinating these components within a single workflow may facilitate procedural efficiency and more consistent maintenance of fracture alignment.

The purpose of this study was to compare perioperative, radiographic, and functional outcomes associated with an integrated workflow combining robot-assisted navigation and mechanically maintained traction vs. conventional manual traction in patients undergoing retrograde intramedullary nailing for AO/OTA 33-A1 and 33-A2 distal femoral fractures.

## Patients and methods

### Study design and patient selection

This single-center retrospective comparative cohort study included patients with distal femoral fractures treated with retrograde intramedullary nailing between January 2021 and December 2024. A total of 58 patients were included. Both surgical workflows were available contemporaneously during the study period. Following discussion of the available surgical options, patients selected either the integrated robot-assisted workflow or the conventional manual-traction workflow. Patients who selected the integrated workflow were assigned to the robot-assisted double reverse traction reduction group (RDR group, *n* = 26), whereas those who selected conventional manual traction were assigned to the conventional manual traction group (CMT group, *n* = 32).

The inclusion criteria were: (1) age ≥18 years with skeletal maturity; (2) an acute traumatic, closed, extra-articular distal femoral fracture classified as AO/OTA 33-A1 or 33-A2; (3) treatment with retrograde intramedullary nailing during the study period; (4) available preoperative anteroposterior and lateral radiographs and/or computed tomography images sufficient for fracture classification; and (5) complete perioperative records and at least 12 months of clinical and radiographic follow-up.

The exclusion criteria were: (1) AO/OTA 33-A3, 33-B or 33-C fractures; (2) open fractures; (3) fractures through bone affected by a primary bone tumor, metastatic disease, multiple myeloma, or another documented focal pathological lesion; (4) periprosthetic distal femoral fractures adjacent to a knee arthroplasty or pre-existing femoral implant; (5) concomitant ipsilateral injuries materially affecting fixation, weight bearing, or functional assessment; (6) active infection involving the operative limb; and (7) severe systemic conditions considered by the anesthesiology and surgical teams to constitute a contraindication to surgery.

Preoperative radiographs and computed tomography images were reviewed to assess fracture morphology, and all fractures were classified according to the AO/OTA classification system. Only extra-articular AO/OTA 33-A1 and 33-A2 fractures were included, and AO/OTA subtype was used to characterize fracture complexity. All included fractures were closed, and none of the patients had a documented diagnosis of osteoporosis or associated injuries. Before implementation of the integrated workflow, the surgical team had previous clinical experience with robot-assisted orthopedic procedures and underwent standardized technical training provided by the manufacturer of the robotic navigation system. All procedures included in the present study were subsequently performed by the same senior orthopedic trauma team under a standardized perioperative management protocol.

### Integrated surgical workflow design

Patients in the RDR group were treated using an integrated surgical workflow combining orthopedic robot-assisted navigation and a double reverse traction reduction frame. The workflow consisted of four standardized stages:

Stage 1: Preoperative planning workflow. Preoperative CT data were used for fracture analysis and surgical path planning. Stage 2: Robotic navigation-assisted positioning workflow. Under fluoroscopic guidance, the robotic system (Tinavi Medical Technologies Co., Ltd., Beijing, China) was used to assist entry-point localization and guidewire placement according to the preplanned trajectory (Figure 1). Stage 3: Continuous traction reduction workflow. The double reverse traction reduction frame (GCQY series, WEGO, Shandong, China) was used to provide continuous longitudinal traction and to assist fracture reduction and maintenance of alignment during fixation. This system provided stable reduction throughout the procedure without reliance on manual traction (Figure 2). Stage 4: Fixation workflow. After satisfactory reduction, retrograde intramedullary nailing was performed in a standard manner. Distal and proximal locking screws were inserted under the robotic system guidance to complete fixation (Figure 3).

**Figure 1:**
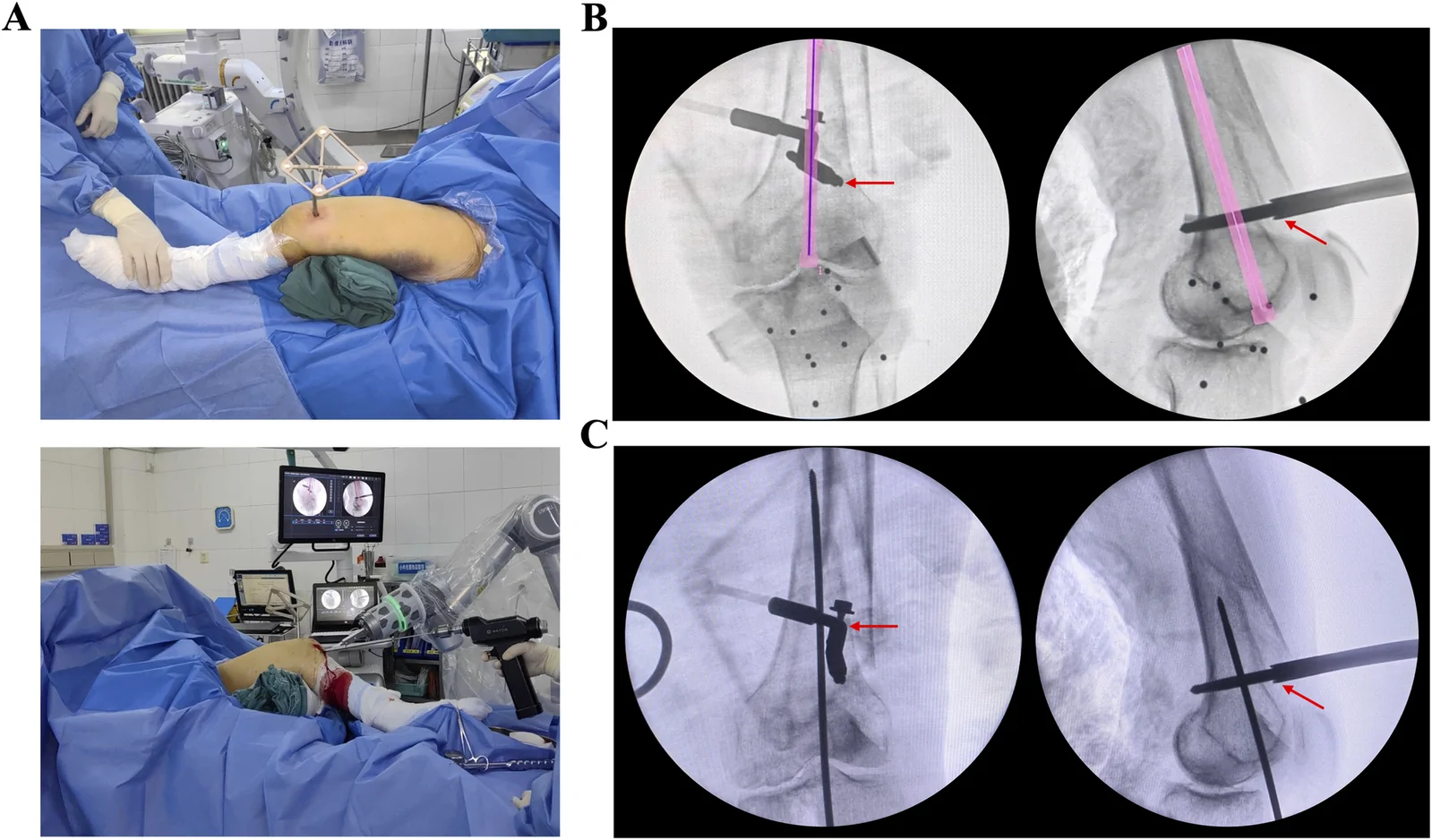
Robot-assisted planning of the entry point and guidewire trajectory for retrograde intramedullary nailing. **(A)** Intraoperative setup of the robotic navigation system. **(B)** Planning of the entry point and guidewire trajectory for retrograde intramedullary nailing using the orthopedic robotic system. The virtual screw indicates the planned guidewire entry point and insertion trajectory. The arrow indicates the tracker. **(C)** Guidewire insertion along the robot-planned trajectory, demonstrating appropriate entry-point position and guidewire direction. The arrow indicates the tracker.

**Figure 2:**
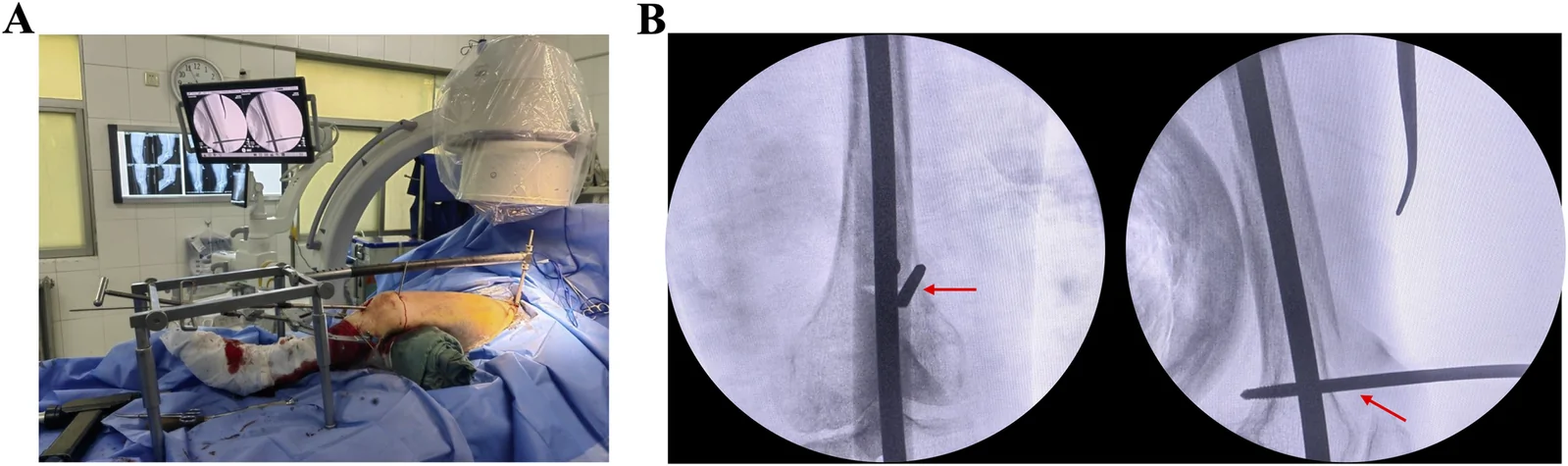
Fracture reduction using the double reverse traction reduction frame. **(A)** Intraoperative setup of the traction reduction frame, with continuous traction applied to assist fracture reduction. **(B)** Intraoperative fluoroscopic image after traction-assisted reduction, demonstrating satisfactory fracture alignment. The arrow indicates the Steinmann pin used to secure the tracker, which also served as a blocking pin to assist fracture reduction.

**Figure 3:**
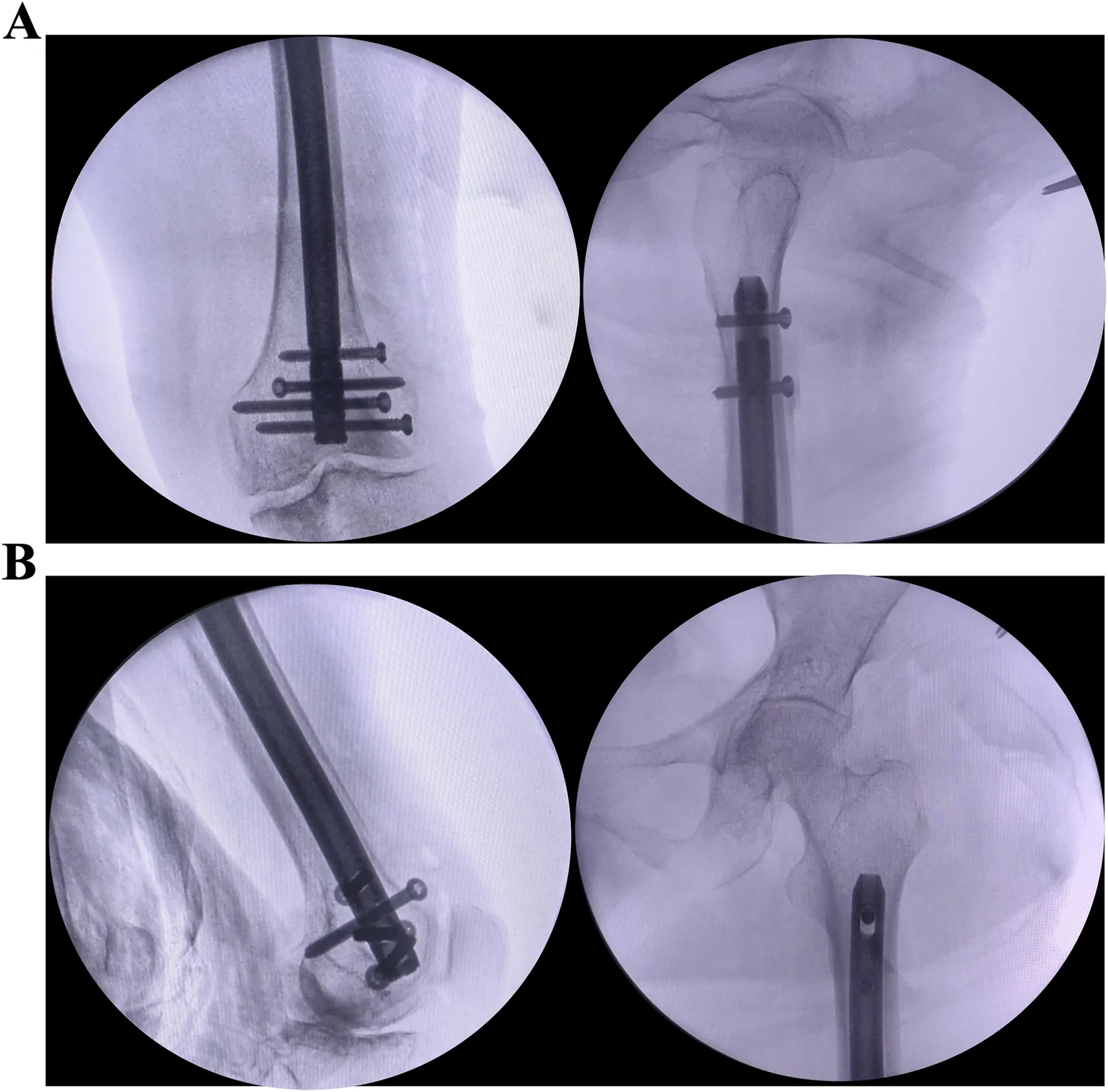
Final fixation after retrograde intramedullary nailing. **(A)** Anteroposterior fluoroscopic image demonstrating fracture alignment, intramedullary nail position, and locking screw placement. **(B)** Lateral fluoroscopic image demonstrating sagittal alignment and implant position.

### Conventional surgical workflow

Patients in the CMT group underwent retrograde intramedullary nailing using a conventional manual traction-assisted workflow. Reduction was achieved using manual traction provided by surgical assistants, and fracture alignment was maintained using fluoroscopic guidance. Implant positioning and guidewire insertion were performed without robotic assistance.

### Perioperative and clinical outcomes

The perioperative outcomes included operative time (min), intraoperative blood loss (mL), and the number of intraoperative fluoroscopic acquisitions. Radiographic outcomes included fracture healing time (weeks) and reduction quality, assessed using residual coronal and sagittal angulation. Functional outcomes included the 12-month knee flexion angle and the 12-month Hospital for Special Surgery (HSS) knee score. Postoperative complications included infection, deep vein thrombosis, delayed union, nonunion, malunion, implant failure, secondary loss of reduction, knee stiffness, and reoperation.

Fracture healing was assessed on serial follow-up radiographs. Radiographic fracture union was defined as bridging callus across at least three cortices. Knee stiffness was defined as knee flexion <90° at the 12-month follow-up. Residual coronal angulation was measured on postoperative anteroposterior radiographs as the angle between the anatomical axes of the proximal and distal femoral segments, whereas residual sagittal angulation was measured analogously on lateral radiographs. Both parameters were independently measured by two assessors.

### Postoperative management

Intravenous antibiotic prophylaxis was routinely administered within the first 24 h after surgery. Low-molecular-weight heparin was initiated subcutaneously on the first postoperative day for prophylaxis against lower-extremity deep vein thrombosis. From the second postoperative day, patients were instructed to begin active and passive in-bed rehabilitation exercises. The timing of weight-bearing was determined individually according to postoperative radiographic evaluation.

Patients underwent regular outpatient follow-up, during which radiographs were obtained to assess fracture healing and alignment. At 12 months postoperatively, knee function was evaluated using the HSS score by two physicians who had not participated in the index procedures. Because treatment allocation could be identifiable from the medical records and radiographic images, complete blinding of the outcome assessors could not be guaranteed.

### Statistical analysis

Continuous variables were expressed as mean ± standard deviation (SD). For normally distributed variables, homogeneity of variance was assessed using Levene's test. Between-group comparisons were performed using the independent-samples Student's t test when variances were equal and Welch's *t* test when variances were unequal. Non-normally distributed continuous variables were compared using the Mann–Whitney U test. For continuous outcomes, between-group effects were additionally expressed as mean differences (MDs) with 95% confidence intervals (CIs), calculated as RDR minus CMT. Categorical variables were presented as numbers and percentages and compared using Pearson's *χ*^2^ test when all expected cell frequencies were ≥5; otherwise, a two-sided Fisher's exact test was used. For postoperative complications, the absolute risk difference (RD) was calculated as the event risk in the RDR group minus that in the CMT group, with corresponding 95% CIs estimated using the Newcombe–Wilson method.

Multivariable linear regression was performed to adjust for potential confounding, with covariates selected *a priori* according to clinical relevance. Regression coefficients, 95% confidence intervals (CIs), and *P* values were estimated using heteroscedasticity consistent HC3 robust standard errors. Model robustness was evaluated by sensitivity analyses. Potentially influential observations were identified using Cook's distance >4/N, and the same models were refitted after their exclusion. In addition, 3,000 nonparametric bootstrap resamples were performed to obtain percentile-based 95% CIs for the estimated effects. All statistical tests were two-sided, and *P* < 0.05 was considered statistically significant.

## Results

### Baseline characteristics

A total of 58 patients with distal femoral fractures were included in this study, with 26 patients in the RDR group and 32 in the CMT group. The RDR group and CMT group were similar in the measured baseline characteristics, including sex, age, height, weight, BMI, injured side, injury mechanism, AO/OTA subtype and time from injury to surgery (*P* > 0.05) (Table 1).

**Table 1:** Baseline demographic and clinical characteristics of patients in the RDR and CMT groups.

Characteristic	RDR group (*n*=26)	CMT group (*n*=32)	Mean difference (95% CI)	*t* or *χ*^2^	*P*
Sex					
Male	14	18	–	–	–
Female	12	14	−	0.034	0.855
Age, years	48.31 ± 15.62	47.56 ± 14.32	0.75 (−7.15 ∼ 8.64)	0.186	0.851
Height, cm	169.42 ± 6.66	170.09 ± 6.43	−0.67 (−4.13 ∼ 2.79)	−0.389	0.699
Weight, kg	68.35 ± 13.86	67.03 ± 10.20	1.31 (−5.01 ∼ 7.64)	0.416	0.679
BMI, kg/m^2^	23.71 ± 4.11	23.26 ± 3.95	0.46 (−1.67 ∼ 2.58)	0.43	0.669
Injury mechanism					
Fall	11	16	–	–	–
Traffic accident	15	16	−	0.341	0.559
Injured side					
Left	16	17	–	–	–
Right	10	15	−	0.414	0.52
AO/OTA fracture subtype					
33-A1	15	20	–	–	–
33-A2	11	12	−	0.139	0.71
Time from injury to surgery, days	3.08 ± 0.89	3.00 ± 0.84	0.08 (−0.38 ∼ 0.53)	0.337	0.737

### Intraoperative workflow efficiency

Operative time was significantly shorter in the RDR group than in the CMT group (97.12 ± 17.33 vs. 132.66 ± 11.71 min; mean difference, −35.54 min; 95% CI, −43.57 to −27.51; *P* < 0.001). Intraoperative blood loss was also lower in the RDR group (290.38 ± 120.02 vs. 451.56 ± 132.28 mL; mean difference, −161.18 mL; 95% CI, −227.66 to −94.69; *P* < 0.001) (Table 2 and Supplementary Figure 1). After adjustment for the prespecified potential confounders, the RDR workflow remained associated with a shorter operative time (adjusted mean difference, −36.11 min; 95% CI, −45.17 to −27.05; *P* < 0.001) and lower intraoperative blood loss (adjusted mean difference, −167.48 mL; 95% CI, −247.29 to −87.66; *P* < 0.001) (Supplementary Table 1). Sensitivity analyses produced estimates that were consistent in direction and similar in magnitude to the primary adjusted estimates (Supplementary Table 2).

**Table 2:** Comparison of operative data between the RDR and CMT groups.

Operative parameter	RDR group (*n*=26)	CMT group (*n*=32)	Mean difference (95% CI)	*t*	*P*
Operative time, min	97.12 ± 17.33	132.66 ± 11.71	−35.54 (−43.57 ∼ − 27.51)	−8.931	<0.001
Blood loss, mL	290.38 ± 120.02	451.56 ± 132.28	−161.18 (−227.66 ∼ − 94.69)	−4.809	<0.001
Fluoroscopic acquisitions	26.15 ± 4.14	38.59 ± 5.51	−12.44 (−14.98 ∼ − 9.90)	−9.81	<0.001

### Fluoroscopic imaging requirements

The number of intraoperative fluoroscopic acquisitions was lower in the RDR group compared with the CMT group (26.15 ± 4.14 vs. 38.59 ± 5.51; mean difference, −12.44 acquisitions; 95% CI, −14.98 to −9.90; *P* < 0.001). This association remained after multivariable adjustment (adjusted mean difference, −12.70 acquisitions; 95% CI, −15.59 to −9.80; *P* < 0.001) (Table 2, Supplementary Table 1 and Figure 1). The association remained consistent in the sensitivity analyses (Supplementary Table 2).

### Fracture healing and radiological outcomes

All patients completed at least 12 months of follow-up. The mean follow-up duration was 15.58 ± 2.06 months in the RDR group and 14.91 ± 2.49 months in the CMT group, with no statistically significant between-group difference (mean difference, 0.67 months; 95% CI, −0.55 to 1.89; *P* = 0.276).

Fracture healing time was shorter in the RDR group than in the CMT group (14.54 ± 4.27 vs. 19.31 ± 4.68 weeks; mean difference, −4.77 weeks; 95% CI, −7.13 to −2.41; *P* < 0.001). Postoperative radiographic assessment showed smaller residual angulation in the RDR group. Residual coronal angulation was 0.85 ± 0.43° in the RDR group compared with 2.84 ± 0.80° in the CMT group (mean difference, −1.98°; 95% CI, −2.31° to −1.65°; *P* < 0.001). Residual sagittal angulation was 1.27 ± 0.81° and 3.54 ± 0.98°, respectively (mean difference, −2.27°; 95% CI, −2.74° to −1.80°; *P* < 0.001) (Table 3 and Supplementary Figure 1).

**Table 3:** Comparison of clinical outcomes between the RDR and CMT groups.

Outcome	RDR group (*n*=26)	CMT group (*n*=32)	Mean difference (95% CI)	*t*	*P*
Reduction quality					
Residual coronal angulation, degree	0.85 ± 0.43°	2.84 ± 0.80°	−1.98° (−2.31° ∼ − 1.65°)	−12.05	<0.001
Residual sagittal angulation, degree	1.27 ± 0.81°	3.54 ± 0.98°	−2.27° (−2.74° ∼ − 1.80°)	−9.474	<0.001
Follow-up time, months	15.58 ± 2.06	14.91 ± 2.49	0.67 (−0.55 ∼ 1.89)	1.099	0.276
Healing time, weeks	14.54 ± 4.27	19.31 ± 4.68	−4.77 (−7.13 ∼ − 2.41)	−4.016	<0.001
12-month knee angle, degree	126.73 ± 13.63°	113.28 ± 17.85°	13.45° (5.16° ∼ 21.74°)	3.163	0.002
12-month HSS scores	89.23 ± 7.38	83.06 ± 6.31	6.17 (2.50 ∼ 9.84)	3.43	0.001

After multivariable adjustment, the RDR workflow remained associated with shorter fracture healing time (adjusted mean difference, −4.65 weeks; 95% CI, −7.20 to −2.10; *P* < 0.001), smaller residual coronal angulation (adjusted mean difference, −1.98°; 95% CI, −2.32° to −1.65°; *P* < 0.001), and smaller residual sagittal angulation (adjusted mean difference, −2.36°; 95% CI, −2.80° to −1.92°; *P* < 0.001) (Supplementary Table 1). Sensitivity analyses showed comparable effect estimates, with the direction and magnitude of the associations remaining broadly consistent with the primary multivariable analyses (Supplementary Table 2). A representative case is shown in Figure 4.

**Figure 4:**
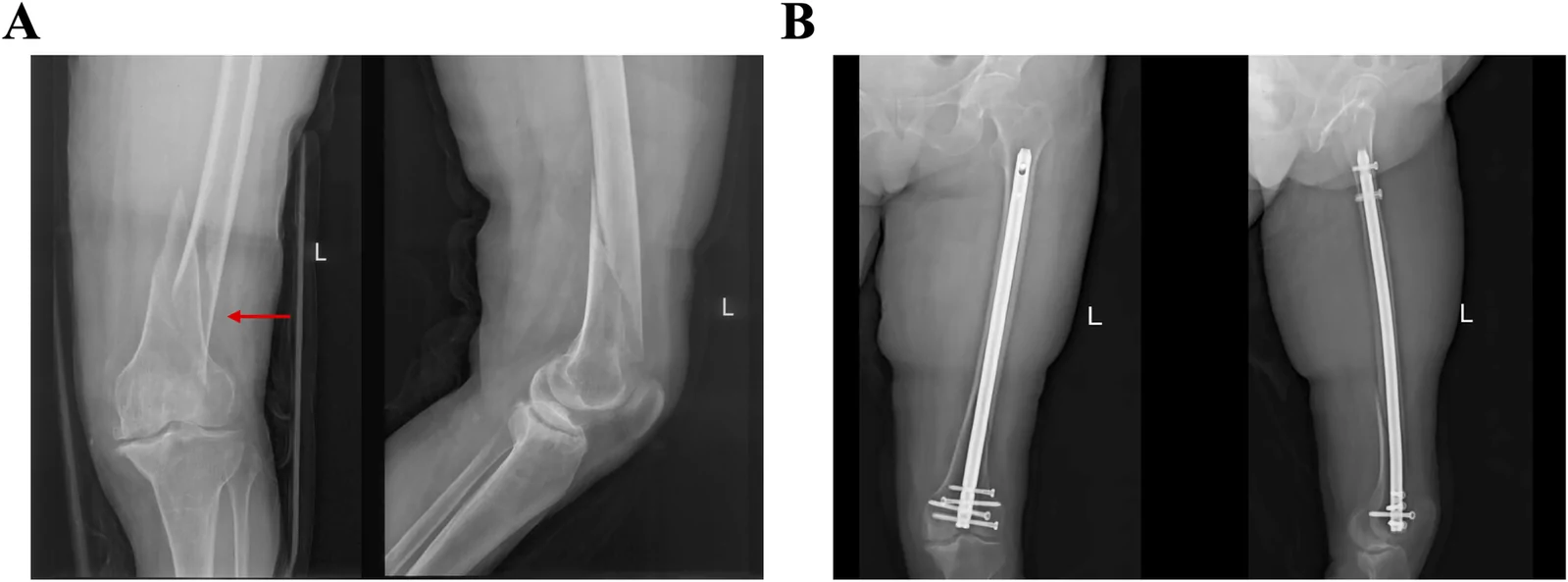
Preoperative and postoperative radiographs of a representative case. **(A)** Preoperative radiograph showing an AO/OTA 33-A1 distal femoral fracture; the arrow indicates the fracture site. **(B)** Postoperative radiograph after retrograde intramedullary nailing using the integrated robot-assisted navigation and mechanical traction workflow, demonstrating postoperative fracture alignment and fixation.

### Functional outcomes

At the 12-month follow-up, knee flexion angle was greater in the RDR group than in the CMT group (126.73 ± 13.63° vs. 113.28 ± 17.85°; mean difference, 13.45°; 95% CI, 5.16° to 21.74°; *P* = 0.002). The 12-month HSS score was also higher in the RDR group (89.23 ± 7.38 vs. 83.06 ± 6.31 points; mean difference, 6.17 points; 95% CI, 2.50 to 9.84; *P* = 0.001) (Table 3 and Supplementary Figure 1). The associations persisted after multivariable adjustment. The adjusted mean difference was 13.47° for knee flexion angle (95% CI, 4.64° to 22.30°; *P* = 0.003) and 6.21 points for the HSS knee score (95% CI, 2.31 to 10.10; *P* = 0.002) (Supplementary Table 1). The sensitivity analyses yielded findings consistent with the primary adjusted analyses (Supplementary Table 2).

### Complications

No cases of infection, nonunion, malunion, implant failure, secondary loss of reduction, or reoperation were recorded in either group during follow-up. Deep vein thrombosis, delayed union, and knee stiffness were observed, but no statistically significant between-group differences were identified for these complications (Table 4). Deep vein thrombosis occurred in 4 of 26 patients (15.4%) in the RDR group and 7 of 32 patients (21.9%) in the CMT group [risk difference (RD), −6.5%; 95% CI, −25.7% to 14.7%; *P* = 0.738]. Delayed union was recorded in 1 of 26 patients (3.8%) and 3 of 32 patients (9.4%), respectively (RD, −5.5%; 95% CI, −20.7% to 10.7%; *P* = 0.620). Knee stiffness was observed in 1 of 26 patients (3.8%) in the RDR group and 5 of 32 patients (15.6%) in the CMT group (RD, −11.8%; 95% CI, −28.2% to 5.6%; *P* = 0.209). All corresponding 95% CIs for the risk differences included zero, indicating substantial uncertainty in the estimated between-group differences.

**Table 4:** Comparison of postoperative complications between the RDR and CMT groups.

Complication	RDR group (*n*=26)	CMT group (*n*=32)	*P*	Risk difference (95%CI)
Deep vein thrombosis	4/26 (15.4%)	7/32 (21.9%)	0.738	−6.5% (−25.7% ∼ 14.7%)
Delayed union	1/26 (3.8%)	3/32 (9.4%)	0.62	−5.5% (−20.7% ∼ 10.7%)
Knee stiffness	1/26 (3.8%)	5/32 (15.6%)	0.209	−11.8% (−28.2% ∼ 5.6%)

No cases of infection, nonunion, malunion, implant failure, secondary loss of reduction, or reoperation were recorded in either group.

## Discussion

Distal femoral fractures remain among the most challenging injuries in orthopedic trauma surgery due to the difficulty in achieving accurate reduction, maintaining stable alignment, and minimizing surgical invasiveness (15). Although retrograde intramedullary nailing is widely accepted as an effective minimally invasive fixation method, its success is highly dependent on intraoperative reduction quality and implant positioning (3–10). In conventional practice, these steps are heavily influenced by manual traction and fluoroscopy-guided adjustments, leading to variability in surgical outcomes (11, 16, 17). In the present retrospective comparative cohort, the integrated workflow combining robot-assisted navigation with a double reverse traction reduction frame was associated with shorter operative time, lower intraoperative blood loss, fewer fluoroscopic acquisitions, shorter fracture healing time, smaller residual coronal and sagittal angulation, and better 12-month knee functional outcomes than conventional manual traction. These associations persisted after adjustment for prespecified measured confounders. Sensitivity analyses yielded associations that were consistent in direction and broadly similar in magnitude to those of the primary multivariable models, supporting the robustness of the observed findings.

Previous studies of orthopedic robotic systems have primarily focused on specific technical steps of intramedullary fixation rather than on the complete process of fracture reduction and fixation. Suero et al. (12) evaluated robotic assistance for entry-point alignment during femoral intramedullary nailing in a cadaveric setting, whereas Panzica et al. (13) investigated robot-assisted distal locking of intramedullary nails. Other studies have explored robotic navigation for fixation of intertrochanteric, femoral shaft, or femoral neck fractures (18–23). Collectively, these studies support the technical feasibility of robotic systems for surgical planning, guidewire positioning, and implant placement, but they do not directly address the challenge of maintaining distal femoral fracture reduction during reaming and nail insertion. Previous studies evaluating double reverse traction reduction frames have focused primarily on their mechanical reduction function. Lian et al. (14) reported the use of such a device during retrograde intramedullary nailing of distal femoral fractures, and other studies have compared double reverse traction with manual traction or traction-table techniques in intertrochanteric, femoral shaft, and other lower-extremity fractures (24–31). These studies suggest that continuous mechanical traction may facilitate closed reduction and decrease reliance on sustained manual traction; however, a traction reduction frame itself does not provide navigation for the entry point or guidewire trajectory. The distinction of the present workflow is therefore the coordinated use of two technologies addressing different procedural requirements. Robot-assisted navigation facilitates spatial planning and guidewire positioning, whereas the double reverse traction reduction frame assists in restoring and maintaining limb length and fracture alignment during fixation. The distinction of the present workflow is therefore the coordinated use of two technologies addressing different procedural requirements. Based on our operative experience, one of the most technically demanding aspects of retrograde intramedullary nailing was not simply obtaining an initial fracture reduction, but maintaining appropriate longitudinal, coronal, and sagittal alignment while the entry point was established, the medullary canal was prepared, and the nail was advanced. With conventional manual traction, an initially satisfactory reduction may be partially lost during subsequent manipulation, reaming, or implant insertion. In the integrated workflow, robotic navigation and mechanical traction addressed different but complementary technical requirements. Robotic navigation primarily facilitated entry-point planning, guidewire positioning, and trajectory control, whereas the double reverse traction reduction frame provided continuous mechanical traction to assist fracture reduction and maintain limb length and coronal and sagittal alignment throughout fixation.

The perioperative findings are generally consistent with the procedural advantages previously reported for robotic navigation and mechanically assisted traction. The integrated workflow was associated with substantially shorter operative time and lower intraoperative blood loss after multivariable adjustment. One possible explanation is that preplanned guidewire positioning together with mechanically maintained reduction reduced the need for repeated manipulation and repositioning. The lower number of fluoroscopic acquisitions observed with the integrated workflow should be interpreted similarly. Robot-assisted planning and more stable maintenance of alignment may decrease the need for repeated fluoroscopic confirmation during guidewire positioning and fracture reduction. The addition of quantitative postoperative alignment measurements provides further information regarding reduction quality. Residual coronal and sagittal angulation were smaller in the integrated-workflow group, and these associations persisted after multivariable adjustment. These findings support better postoperative coronal and sagittal alignment within the study population. Continuous mechanical traction may help maintain length and alignment while the implant is introduced, whereas navigation may facilitate controlled guidewire positioning. Fracture healing time was also shorter in association with the integrated workflow, while knee flexion and HSS knee scores at 12 months were higher. Maintenance of alignment, less repeated manipulation, and reduced operative disturbance could theoretically provide conditions favorable to fracture healing and postoperative rehabilitation. Similarly, improved alignment may contribute to restoration of knee mechanics. However, the present study was not designed to test these variables as mediators, and no causal pathway between the surgical workflow, radiographic alignment, fracture healing, and functional recovery can be established. The functional findings should therefore be interpreted as clinical associations accompanying the integrated workflow rather than direct consequences of any single technical component.

No statistically significant between-group differences were observed in the recorded postoperative complication rates. However, given the limited sample size and small number of events, these findings should not be interpreted as evidence of equivalent safety. Larger prospective studies are needed to more reliably evaluate uncommon adverse outcomes and comparative safety.

Compared with conventional manual traction-assisted surgery, the integrated workflow may reduce the need for continuous manual assistance and repeated intraoperative repositioning. Conventional manual traction is often intermittent and dependent on coordination among surgical personnel, which may affect the stability of fracture reduction (11). In contrast, continuous mechanical traction provided by the double reverse traction reduction frame may help maintain fracture alignment during fixation, while robot-assisted navigation facilitates procedural planning and guidewire positioning (14, 24). These complementary functions may contribute to a more standardized intraoperative workflow; however, operator dependence and inter-surgeon variability were not directly evaluated in the present study.

The surgical workflow in this study was designed using a staged integration strategy to coordinate robotic navigation with mechanically assisted fracture reduction. Initially, robotic navigation was utilized to determine and access the optimal entry point for retrograde intramedullary nailing. Subsequently, a double reverse traction device was applied to achieve fracture reduction, to minimize potential mechanical interference with the robotic arm and facilitate procedural continuity. In our configuration, the double reverse traction reduction frame applied counter-traction via skeletal traction pins placed in the distal femur (or tibial tuberosity depending on fracture pattern). For the RDR group, the Steinmann pin for the robotic tracker was placed in the ipsilateral femoral condyle. This configuration was selected for several functional and anatomical reasons. First, Positioning both the navigation reference and the guidewire entry region within the same distal bone segment was intended to maintain a stable spatial relationship during intraoperative manipulation. Second, the relatively broad structure of the femoral condyle provides sufficient safe corridors, allowing avoidance of the medullary canal expansion path as well as planned locking screw trajectories. Third, depending on the direction of fracture displacement, the Steinmann pin can serve as a temporary blocking pin, which may be placed in either the medial or lateral condyle to assist in fracture reduction. Finally, the same Steinmann pin can function as a joystick, facilitating intraoperative correction of rotational malalignment. Despite the surgeons’ previous experience with robot-assisted procedures, implementation of the integrated workflow involved an additional learning process. Particular attention was required during traction-frame setup and in coordinating navigation-assisted guidewire placement with mechanically maintained fracture reduction. Previous experience with robotic surgery and standardized manufacturer-provided training may facilitate adoption of the navigation component; however, effective integration of robotic navigation and mechanical traction also requires familiarity with the combined workflow and close coordination among surgeons, assistants, nursing staff, and technical personnel.

The applicability of the present findings should also be considered in relation to patient selection. The age threshold of 18 years in this study was intended to define an adult, skeletally mature population and should not be interpreted as indicating that retrograde intramedullary nailing is the preferred treatment for all patients aged ≥18 years. Treatment of younger adults with distal femoral fractures should be individualized according to skeletal maturity, AO/OTA fracture pattern, displacement, soft-tissue condition, bone quality, associated injuries, and functional requirements. Similarly, the double reverse traction reduction frame should not be regarded as universally applicable to distal femoral fractures. Its use may be unsuitable or require particular caution in intra-articular fractures requiring direct articular reconstruction, severe open fractures or extensive soft-tissue injury, infection around potential pin-placement sites, the presence of prostheses or previous implants that interfere with safe pin placement, severe osteoporosis that compromises traction-pin purchase, skeletal immaturity, associated vascular injury, or fracture configurations in which rotation, coronal displacement, or a short distal fragment cannot be adequately controlled by longitudinal traction alone. Alternative reduction techniques should be used when satisfactory restoration of length, rotation, and alignment cannot be safely achieved or maintained with the traction frame.

The potential procedural advantages of the integrated workflow must also be balanced against its resource and implementation requirements. The technique requires access to both an orthopedic robotic navigation platform and a double reverse traction reduction frame, together with equipment maintenance and sterilization, operating-room space, device preparation, robotic registration, image acquisition, path planning, and coordination among surgical, nursing, imaging, and technical personnel. These requirements may limit immediate implementation in community hospitals, low-volume centers, and resource-limited settings. The present study did not prospectively record device-specific setup time, staff workload, total operating-room occupancy, or patient-level costs, and no formal cost-effectiveness analysis was performed. Therefore, the economic feasibility of the integrated workflow remains to be established.

Several limitations should be acknowledged. First, the retrospective, non-randomized design may have introduced selection bias and residual confounding despite multivariable adjustment and sensitivity analyses. Second, the relatively small sample size and single-center setting limit statistical precision and generalizability. Third, only closed, extra-articular AO/OTA 33-A1 and 33-A2 fractures treated with retrograde intramedullary nailing were included. Fourth, because the integrated workflow was evaluated as a whole, the independent contributions of robotic navigation and the traction reduction frame could not be determined. In addition, direct measurements of navigation accuracy, rotational alignment, traction force, fluoroscopy duration, and radiation dose were unavailable. Complications were identified retrospectively, and the follow-up duration was insufficient to assess long-term outcomes. Future studies should include larger prospective multicenter cohorts with longer follow-up and, where feasible, study designs that separately evaluate the contributions of robotic navigation and mechanically assisted traction. In addition, standardized contralateral limb imaging was not prospectively obtained, and femoral length discrepancy was not quantitatively assessed in the present study. Future prospective studies should incorporate standardized bilateral radiographs or other validated measurement methods to evaluate restoration of femoral length.

## Conclusion

In this retrospective comparative cohort of patients with AO/OTA 33-A1 and 33-A2 distal femoral fractures, robot-assisted navigation combined with a double reverse traction reduction frame was associated with shorter operative time, lower blood loss, fewer fluoroscopic acquisitions, improved postoperative alignment, and better 12-month knee function compared with conventional manual traction.

## Data Availability

The raw data supporting the conclusions of this article will be made available by the authors, without undue reservation.
