# Supplementary material for: Robot-assisted navigation combined with a double reverse traction reduction frame for retrograde intramedullary nailing of extra-articular distal femoral fractures: a retrospective comparative cohort study
Source: Front Med (Lausanne). 2026 Oct 19;13:1929866. doi: 10.3389/fmed.2026.1929866 (PMC13724628; doi:10.3389/fmed.2026.1929866)
Supplement: Supplementary file 1 [file Table1.docx]

Supplementary Table 1. Multivariable Linear Regression Analyses of the Primary Continuous Outcomes

|  | Adjusted unstandardized coefficient, B (RDR minus CMT) | 95%CI | P | Adjusted R² |
| --- | --- | --- | --- | --- |
| Operative time, min | −36.11 | −45.17~−27.05 | <0.001 | 0.58 |
| Blood loss, mL | −167.48 | −247.29~−87.66 | <0.001 | 0.299 |
| Fluoroscopic acquisitions | −12.7 | −15.59~−9.80 | <0.001 | 0.592 |
| Reduction quality | |  |  |  |
| Residual coronal angulation, degree | −1.98° | −2.32°~−1.65° | <0.001 | 0.698 |
| Residual sagittal angulation, degree | −2.36° | −2.80°~−1.92° | <0.001 | 0.635 |
| Healing time, weeks | −4.65 | −7.20~−2.10 | <0.001 | 0.179 |
| 12-month knee angle, degree | 13.47° | 4.64°~22.30° | 0.003 | 0.092 |
| 12-month HSS scores | 6.21 | 2.31~10.10 | 0.002 | 0.16 |

Covariates were selected a priori according to clinical relevance and included age, body mass index, AO/OTA fracture subtype, injury mechanism, and time from injury to surgery.
