# Supplementary material for: Robot-assisted navigation combined with a double reverse traction reduction frame for retrograde intramedullary nailing of extra-articular distal femoral fractures: a retrospective comparative cohort study
Source: Front Med (Lausanne). 2026 Oct 19;13:1929866. doi: 10.3389/fmed.2026.1929866 (PMC13724628; doi:10.3389/fmed.2026.1929866)
Supplement: Supplementary file 2 [file Table2.docx]

Supplementary Table 2. Sensitivity Analyses of the Multivariable Linear Regression Models for the Primary Continuous Outcomes

|  | Adjusted unstandardized coefficient, B (RDR minus CMT) | 95%CI | P | Bootstrap 95%CI |
| --- | --- | --- | --- | --- |
| Operative time, min | −39.62 | −48.08~−31.16 | <0.001 | −44.87~−27.92 |
| Blood loss, mL | −171.94 | −240.22~−103.66 | <0.001 | −238.08~−89.62 |
| Fluoroscopic acquisitions | −12.71 | −15.57~−9.86 | <0.001 | −15.42~−10.04 |
| Reduction quality | |  |  |  |
| Residual coronal angulation, degree | −1.96° | −2.28°~−1.63° | <0.001 | −2.30~−1.66 |
| Residual sagittal angulation, degree | −2.62° | −2.99°~−2.24° | <0.001 | −2.78~−1.95 |
| Healing time, weeks | −4.57 | −6.61~−2.52 | <0.001 | −7.07~−2.45 |
| 12-month knee angle, degree | 14.58° | 6.35°~22.82° | <0.001 | 5.38~21.63 |
| 12-month HSS scores | 7.77 | 4.36~11.17 | <0.001 | 2.66~9.70 |

Covariates were selected a priori according to clinical relevance and included age, body mass index, AO/OTA fracture subtype, injury mechanism, and time from injury to surgery.
